# Music From the Very Beginning—A Neuroscience-Based Framework for Music as Therapy for Preterm Infants and Their Parents

**DOI:** 10.3389/fnbeh.2018.00112

**Published:** 2018-06-05

**Authors:** Friederike Barbara Haslbeck, Dirk Bassler

**Affiliations:** Department of Neonatology, University Hospital Zurich and University of Zurich, Zurich, Switzerland

**Keywords:** early auditory experience, brain development, music, preterm infant, parents

## Abstract

Human and animal studies demonstrate that early auditory experiences influence brain development. The findings are particularly crucial following preterm birth as the plasticity of auditory regions, and cortex development are heavily dependent on the quality of auditory stimulation. Brain maturation in preterm infants may be affected among other things by the overwhelming auditory environment of the neonatal intensive care unit (NICU). Conversely, auditory deprivation, (e.g., the lack of the regular intrauterine rhythms of the maternal heartbeat and the maternal voice) may also have an impact on brain maturation. Therefore, a nurturing enrichment of the auditory environment for preterm infants is warranted. Creative music therapy (CMT) addresses these demands by offering infant-directed singing in lullaby-style that is continually adapted to the neonate’s needs. The therapeutic approach is tailored to the individual developmental stage, entrained to the breathing rhythm, and adapted to the subtle expressions of the newborn. Not only the therapist and the neonate but also the parents play a role in CMT. In this article, we describe how to apply music therapy in a neonatal intensive care environment to support very preterm infants and their families. We speculate that the enriched musical experience may promote brain development and we critically discuss the available evidence in support of our assumption.

“The first time I sang “Incy wincy spider” for Rafael was when it became clear that his early arrival would be inevitable. My body, my womb, became a ticking bomb. The feeling was unbearable, and I felt so sorry for him. Through his movements, I could feel he sensed the tension, so I desperately needed something to soothe him. We needed a “happy song” to give us strength and courage. So one night with my tears falling, I started to sing “Incy wincy” for him, and it magically made me smile. From then on, I sang this song for him whenever I could—during the CTGs and many nights on the hospital terrace. When I could finally hold him during the kangaroo care, I sang “Incy wincy” again, and he seemed to react, he seemed to remember. The music brought smiles to the tears. This lovely nursery rhyme had become the messenger of emotions when words failed me. It could convey positive vibrations even when I was crying, and it could lighten up Rafael, and soon we could see his first smile in the NICU mirror. He enjoyed the song, even more, when it was accompanied by the monochord: his face lit up, his eyes moved, he would smile, and soon his oxygen level went up. It was magic. I still sing “Incy wincy” for him.” (Mother of a preterm infant)

## Early Auditory Experiences

The sense of hearing is already developed before birth. The fetus responds to sound at least as early as at 25–27 weeks of gestational age (Hepper and Shahidullah, 1994; Clark-Gambelunghe and Clark, 2015; Monson et al., 2018). At this age, peripheral auditory inputs may already reach the auditory cortex (Jardri et al., 2008; Mahmoudzadeh et al., 2013). Sounds *in utero* are characterized by low volume and low-frequency since maternal tissue and amniotic fluid function as a filter for high-frequency sounds (Graven, 2000). Internally generated sounds originate in the mother’s organs (e.g., whooshing sound of the placenta, rhythmic heartbeat), her voice and her movements. They are of highly musical nature. Heartbeat is rhythmic, and the fetus primarily hears the musical parameters of speech: melody, rhythm, prosody, phonemes and pitch contour of the maternal voice and external voices (Moon et al., 2013; Partanen et al., 2013; Philbin, 2017). Especially prosody transfers emotion, intention and grammatical function that is, e.g., needed for later language differentiation. Prosody may be the most multisensory and comprehensible part of language development and languages across cultures (Huotilainen, 2013; Moon et al., 2013). The developing fetus experiences these sounds already in an emotional, multimodal and vibro-acoustic manner. The fetus hears the voices originating from social activity in conjunction with maternal movements, maternal vocal vibration, vestibular emotion, circadian cycles and hormonal release that crosses the placenta (Moon, 2017).

After birth, newborns seem to recognize and prefer the maternal voice over other female voices (Kisilevsky et al., 2003, 2009). Even the mother’s tongue appears to be favored compared to other languages by neonates (Lecanuet and Schaal, 1996; Kisilevsky et al., 2009; McMahon et al., 2012). This recognition and the possibility of discrimination suggest that the maternal voice plays a predominant role in the development of hearing and that auditory learning and memory starts already before birth. Social behavior such as first patterns of communication and attachment may develop through this early acoustic experience (Filippa et al., 2017).

Brain development is linked to early auditory experience, as demonstrated by both human and animal studies (Chang and Merzenich, 2003; Dahmen and King, 2007; de Villers-Sidani et al., 2008). Auditory brain plasticity and the development of the auditory cortex are heavily dependent on the quality of hearing experience (Yan, 2003). In a prospective animal study, Angelucci et al. (2007) showed that exposure to music in mice could facilitate the differentiation of new neurons and the production of nerve growth factor in brain areas like the hippocampus, hypothalamus and cortical regions. Studies in music and neuroscience demonstrate that music promotes neurobiological processes, modulates synaptic plasticity, neuronal learning, and readjustment in the human brain (Abbott, 2002; Rickard et al., 2005; Sacks, 2007). Music activates various limbic and paralimbic structures. It seems that this activation may enhance psychological and physiological health (Koelsch, 2010, 2014). Particularly in newborns music promotes the neuronal activation and many researchers suggest that musical learning already starts prior birth (Huotilainen and Näätänen, 2010; Perani et al., 2010).

## Premature Birth From An Auditory Perspective

With premature birth, the intrauterine sheltering environment perfect for fetal growth and maturation is abandoned too early. Besides other stressful experiences such as the separation from the mother and painful procedures, preterm infants must cope with the unusual sound environment of an intensive care unit. Preterm infants are now exposed to high-frequency, loud, and unpredictable auditory stimuli from various machines and monitors. In 1997, the American Academy of Pediatrics recommended that sound levels not exceed 45 dB for neonatal intensive care units (NICUs; American Academy of Pediatrics, 1997). However sound levels in NICUs are often in the range of 50–80 dB, sometimes reaching peaks of 120 dB (Williams et al., 2007; Darcy et al., 2008). Two narrative reviews (Morris et al., 2000; Wachman and Lahav, 2011) suggest that high and stressful noise may have adverse short-term effects on the cardiovascular and respiratory systems of preterm infants, e.g., increased oxygen consumption, apnea and hypoxemia.

Preterm infants are particularly sensitive to noise because their auditory system and brain development are in a critical, vulnerable and fast period of growth. Animal studies suggests that noise can negatively affect the development of auditory-related functioning of the cortex, spatial localization and vocal learning (Marler et al., 1973; Philbin et al., 1994; Chang and Merzenich, 2003). Thus, the overwhelming auditory neonatal intensive care environment is assumed to interfere with the short- and long-term neurobehavioral development in preterm infants (Perlman, 2001; Wachman and Lahav, 2011). Stressful noise can further initiate stress responses in preterm infants. The sympathetic autonomic nervous system is activated, along with the hypothalamic-pituitary-adrenal axis of the endocrine system, known as “fight or flight” reaction (Anderson and Patel, 2018). This response may use energy reserves that are crucial for brain development in preterm infants. Animal studies further suggest that frequent stress exposure may lead to a loss of excitatory synapses in the prefrontal cortex and the hippocampus. Additionally, an increase in the number of dendrites and synaptic spines in the amygdala, and even neuronal death is reported (Radley and Morrison, 2005; Anderson and Patel, 2018). These parts of the brain are crucial for learning, decision-making, and regulation of emotions. Therefore, it is not surprising that preterm infants have a high incidence of long-term neurobehavioral problems. Problems include cognitive and behavioral deficits as well as problems of executive function such as control of attention, working memory, reasoning and strategic planning (Woodward et al., 2006; Wehrle et al., 2016).

Besides the noise, painful procedures and the separation from the mother can augment the stress response in preterm infants (Udry-Jørgensen et al., 2011). Conversely, as shown in animal studies, increased early nurturing social contact with the mother (e.g., pup licking and grooming) reduced stress in the animal babies and had long-term neurodevelopmental benefits (Weaver et al., 2004; Champagne and Curley, 2009). Not only preterm infants but also their parents experience many challenges and face concerns that can induce a stress response. The sudden and unexpected end of pregnancy, the psychological trauma of premature birth, and the uncertainty of the infant’s future can evoke feelings of fear, guilt, loss and grief (Jotzo and Poets, 2005; Flacking et al., 2007; Roque et al., 2017). These reactions may increase parental stress, may also adversely impact the stress-coping behavior of their newborn and the formation of a secure attachment (Borghini et al., 2006; Forcada-Guex et al., 2006; Korja et al., 2011).

Conversely, auditory deprivation (the lack of meaningful auditory stimulation such as the regular intrauterine rhythms of the maternal heartbeat and the mother’s voice) may also impact brain maturation and subsequent speech and language acquisition in preterm infants. The deprivation occurs at a particularly crucial period for neural wiring and subsequent neurodevelopment (Hepper and Shahidullah, 1994; Moon and Fifer, 2000; DeRegnier et al., 2002). Animal studies show that environmental acoustic inputs in early life largely influence the functional development of the auditory system and that acoustic deprivation alters the development of the auditory cortex (Klinke et al., 2001; Iyengar and Bottjer, 2002). In a prospective cohort study with 136 preterm infants (<30 weeks gestation either assigned to the open ward unit or private rooms). Pineda et al. (2014) showed that neonates in quiet private rooms had less brain maturation at term-equivalent age, lower language scores and a trend toward lower motor scores than neonates cared for in a busy open ward.

To summarize, appropriate auditory stimulation and social contact appear to be critical for brain maturation in early life. This process already starts *in utero*, continues into the newborn period and lasts through early neonatal life. Musical learning also occurs before birth. Music activates limbic and paralimbic regions in the human brain. This engagement may enhance psychological and physiological health which is particularly crucial following preterm birth as preterm infants are at risk for neurodevelopmental impairment and the plasticity of auditory regions and cortex development are heavily dependent on the quality of auditory experiences (Yan, 2003; Anderson and Patel, 2018).

## Music as Therapy for Preterm Infants

Based on the described potential adverse effects of auditory overstimulation and sensory deprivation on preterm infants’ neurobehavioral development the importance of providing an adequate environment becomes obvious. Reduction in noise levels in the NICU is warranted to reduce adverse neonatal outcomes and to promote growth. However, more research is needed to be able to provide specific recommendations for the management of sound reduction in neonatal care as outlined by a Cochrane review (Almadhoob and Ohlsson, 2015). Focusing exclusively on reducing sound levels in the NICU may not be a sufficient solution. As we have described, sensory deprivation may also negatively impact on neurodevelopment (Jobe, 2014). Adequate stimulation for the development of the preterm brain is thus recommended (Jobe, 2014; Shoemark et al., 2015). Music interventions—as exemplified by the case vignette above—may be appropriate and are the focus of growing interdisciplinary research activities. They may attenuate the stress response in preterm infants and provide environmental enrichment by meaningful auditory stimulation and social contact (Shoemark et al., 2015; Anderson and Patel, 2018; Haslbeck and Stegemann, 2018).

Creative music therapy (CMT) for preterm infants and their parents is an individualized, interactive, resource- and needs-oriented music therapy approach that is based upon “Nordoff-Robbins music therapy” (Nordoff and Robbins, 1977, 2012). The Nordoff-Robbins approach implies the unique qualities of music as therapy: enhancing communication, supporting change, and enabling people to live more resourcefully and creatively to promote personal growth, health and development (Haslbeck, 2004, 2013). It has been adapted to address the specific needs of preterm infants and their family within the NICU setting (Haslbeck, 2004, 2013). CMT assumes that almost everyone is sensitive and can respond to music, no matter how ill or disabled or even premature an infant is (Nordoff and Robbins, 1977). The non-invasive potential of CMT for pre-linguistic communication allows even vulnerable, severely affected individuals like preterm infants to become “active” rather than being assigned a solely receptive and passive role. Moreover, the focus of CMT involving preterm infants and their parents is on creating an individual relationship with the newborn. It happens through music, as well as by facilitating the relationship with parents to support the newborn “coming into being,” (Aldridge and Aldridge, 1996) the parents into parenthood, and the triad into bonding.

Within the concept of CMT the music therapist assesses the “music” of the preterm infant (the breathing pattern—the most fundamental rhythm of a human being—in conjunction with the newbornś facial expressions and gesticulations) and transforms it into infant-directed humming. The humming is continually tailored to the newborn’s needs; e.g., when the neonate’s eyebrows lift, the music therapist steers the melody upward. Conversely, when the newborn fusses, the music therapist moves oppositely; e.g., bringing the tune downwards and slowing its tempo to soothe the baby with sedative musical parameters (Loewy et al., 2005). The infant-directed humming or singing is as simple as possible since preterm infants can quickly be overwhelmed by auditory stimulation. The music therapist is trained to hum with fluency, using rich overtones in a lullaby and infant-directed style, keeping the music calm, simple, predictable and repetitive as recommended in guidelines on music therapy in the NICU (Hanson-Abromeit et al., 2008). Stern’s theory of affect attunement (Stern, 2010) as well as the theory of communicative musicality by Malloch and Trevarthen (2009), emerged in a qualitative study of CMT as sensitizing concepts (Haslbeck, 2014). We analyzed video footage of 122 music therapy sessions with 18 preterm infants (and their parents) with a broad range of social backgrounds and diagnoses in a qualitative grounded theory based study (Haslbeck, 2014). The micro-analysis of the videotaped sessions suggests that finely-attuned and entrained infant-directed singing offers the potential for preterm infants to engage in communicative musicality. This experience may help the infants to get back in tune, in rhythm, in mutual regulation, in self-synchrony, and in interactional synchrony, even in an overwhelmingly-arrhythmic intensive care environment (Haslbeck, 2013, 2014).

## Family-Integrated Approach to Empower Parents

CMT in neonatal care is a family-integrated approach in which each family is encouraged to find their innate way of relating to their newborn through music. The parents, if available and willing, are involved and integrated individually in the therapeutic process; e.g., by providing music therapy during skin-to-skin holding with the baby and by supporting and encouraging them to sing or speak to their newborn to foster an intuitive parent-infant interaction. To honor the family’s culture and preferences, the music therapist assesses the parents’ musical heritage, favorite musical style and or song(s) (Loewy, 2015). Skin-to-skin contact is now a standard routine in many neonatal care units and it may be the most natural way to deliver music to the preterm infant in a multi-sensory and family-integrated manner (Gooding et al., 2011; O’Brien et al., 2013). As demonstrated in a qualitative study, CMT may empower parents, by enhancing their well-being, self-confidence, and quality of interactions with their newborn through music (Haslbeck, 2014).

When offering music therapy during kangaroo care, the music therapist mainly uses a monochord as accompanying instrument. A monochord is a single-stringed wooden instrument designed for the therapeutic purpose of generating relaxing sounds and vibro-acoustic stimulation to allow for the replication of deep live womb sounds. The monochord is placed next to the kangaroo care chair, so that the parents can touch the instrument with their elbow and feel the instrument’s relaxing vibrations. Since many parents are stressed during kangaroo care, partially due to post-traumatic reactions, the relaxing sound of the monochord may be an opportunity for parents and neonates to efficiently calm down, relax and perceive each other more intensely (Lee et al., 2012).

## Evidence for Music Therapy in Neonatal Care

Several systematic reviews suggest that music stimulation may have beneficial effects on physiologic parameters, behavioral states, sleep quality, oral feeding, and weight gain in preterm infants (Hartling et al., 2009; Hodges and Wilson, 2010; Haslbeck, 2012; Standley, 2012; van der Heijden et al., 2016; Anderson and Patel, 2018). For instance, a systematic integrative review with 43 included studies demonstrates that music stimulation may support pacification and stabilization in preterm infants (more stable heart rate, oxygen saturation, and behavioral states; Haslbeck, 2012). This is in line with findings of an updated systematic review on possible benefits of music interventions on preterm infant’s well-being. van der Heijden et al. (2016) included 20 randomized controlled trials (RCTs) involving 1128 participants who received music stimulation in the NICU. They showed that music may improve neonates’ sleep, heart rate, feeding and sucking outcomes. Additionally, parent-infant interaction may be enhanced (Haslbeck, 2012). A recently updated systematic review encompassing 964 neonates participants and 266 parent participants focused exclusively on well-experienced therapists as providers of the music intervention and included only RCTs. The meta-analyses confirmed a favorable effect of music therapy on neonate respiratory rate (mean difference, −3.91/min, 95% confidence interval, −7.8 to −0.03) and on maternal anxiety (standardized mean difference, −1.82, 95% confidence interval, −2.42 to −1.22; Bieleninik et al., 2016). However, the systematic review could not confirm or refute beneficial effects on other physiologic and behavioral outcomes. Considerable heterogeneity in study populations, interventions and outcomes did prevent definite conclusions on the impact of music therapy in preterm infants in all the reviews (Hartling et al., 2009; Hodges and Wilson, 2010; Haslbeck, 2012; Standley, 2012; Bieleninik et al., 2016; van der Heijden et al., 2016; Anderson and Patel, 2018). Stress reduction and their short-and long-term consequences on preterm infants and their primary caregivers as well as neurobehavioral short- and long-term outcomes have rarely been evaluated in clinical studies and systematic reviews. Other issues that are crucial for providing more comprehensive and rigorous evidence-based recommendations are hardly addressed, such as the impact of various musical and vocal stimulation approaches.

## Conclusion and Implications for Practice and Research

This manuscript provides a perspective on the therapeutic use of music in a neonatal care setting. We provide evidence supporting our assumption why and how music may be used to support very preterm infants and their families. We assume that a family-integrated musical experience may reduce stress, induce meaningful sensory stimulation, nurture interactions contributing to the bonding process and may thereby improve neurodevelopment from the very beginning.

In order not to overwhelm preterm infants and their parents, music interventions should best be performed live by a trained music therapist. It appears crucial to continuously tailor the music to the changing individual needs of both, the newborns and the parents in the sense of family-integrated care (Loewy et al., 2013; Filippa et al., 2017; Anderson and Patel, 2018). Especially in times of increasing technical options to play music, the interactive characteristics of live lullaby singing—nurturing, caring, calming, connecting—should not be underestimated (Haslbeck, 2014). Indeed, neurobehavioral development may accelerate by nourishing social learning experiences and human interaction, as opposed to machine-based stimulation (Patel, 2008; Huotilainen and Näätänen, 2010).

Future trials should address clinically relevant outcomes of music therapy for both, preterm infants and their parents, with a rigorous design. These trials should be of high methodological quality and adequately powered. To date, a significant gap in our understanding of music interventions has been the lack of data examining the effects over time. RCTs should systematically investigate short- and long-term effects of music interventions on brain function and development as well as parental well-being. Such a trial is currently conducted in Switzerland (Haslbeck et al., 2017). Of particular interest are the evaluation of cortical gray and white matter volumes, patterns of functional connectivity, measures of language development and executive functions. Rigorous studies evaluating stress levels are also warranted, e.g., by objectively measuring hypothalamic-pituitary-adrenal activation/regulation (cortisol and *β*-endorphins) and bonding behavior in neonates and parents with and without music (Anderson and Patel, 2018). The latter focus appears to be especially promising since a systematic review had shown that cortisol reduction is one of the most reliable effects of music therapy (Koelsch and Stegemann, 2012). Further evaluating music as a therapeutic option in the NICU will provide new insights into the potential of music on enhancing brain function and development. Music therapy may become a low-cost, low-risk family-integrated standard intervention for preterm infants and their parents and may be found to support neurobehavioral development, well-being and secure attachment in this vulnerable group from the very beginning.

## Author Contributions

FBH wrote the article. DB reviewed the article.

## Conflict of Interest Statement

The authors declare that the research was conducted in the absence of any commercial or financial relationships that could be construed as a potential conflict of interest.
